# Ring finger protein 43 associates with gastric cancer progression and attenuates the stemness of gastric cancer stem-like cells via the Wnt-β/catenin signaling pathway

**DOI:** 10.1186/s13287-017-0548-8

**Published:** 2017-04-26

**Authors:** Yunhe Gao, Aizhen Cai, Hongqing Xi, Jiyang Li, Wei Xu, Yanmei Zhang, Kecheng Zhang, Jianxin Cui, Xiaosong Wu, Bo Wei, Lin Chen

**Affiliations:** 1 Department of General Surgery, Chinese PLA General Hospital, Beijing, 100853 China; 2 School of Medicine, Tsinghua University, Beijing, 10084 China

**Keywords:** Ring finger protein 43, Gastric cancer, Stem cells, Stem-like property

## Abstract

**Background:**

Ring finger protein 43 (RNF43) is a member of the transmembrane E3 ubiquitin ligase family that was originally found in stem cells and plays important roles in tumor formation and progression. Our previous study indicated that RNF43 might be a tumor suppressor protein in gastric cancer. Given its antagonistic relationship with leucine-rich repeat-containing G-protein-coupled receptor 5 (Lgr5), one of the gastric cancer stem cell markers, investigation of the potential role of RNF43 in gastric stem cancer cells is necessary.

**Methods:**

Immunohistochemistry staining, western blot analysis, and quantitative reverse transcription polymerase chain reaction were used to determine the mRNA and protein expression level of RNF43 and other Wnt pathway factors. Gastric cancer stem-like cells were obtained from gastric cancer tumor and cell lines by tumorsphere culture. The adeno-associated virus system was used to upregulate RNF43 expression in cancer cells. Functional experiments including tumorsphere formation, chemotherapy resistance, surface marker detection, and tumor xenograft assay were performed to measure stem-like properties in gastric cancer stem-like cells after RNF43 overexpression.

**Results:**

RNF43 loss was significantly associated with TNM stage, distant metastasis, and Lauren classification, and predicted worse prognosis in gastric cancer patients. RNF43 expression was even lower in tumorspheres derived from tumor tissues or cell lines compared with adherent cancer cells and normal gastric cells. Overexpression of RNF43 in gastric cancer cells impaired their stem-like properties, including sphere formation ability, chemoresistance in vitro, and tumorigenicity in vivo. Moreover, Wnt pathway-related proteins were decreased in RNF43-overexpressing cells, while Wnt pathway activators could reverse the trend to some extent.

**Conclusions:**

Our findings indicated that RNF43 might not only participate in gastric cancer progression, but also attenuate the stemness of gastric cancer stem-like cells through the Wnt/β-catenin pathway.

**Electronic supplementary material:**

The online version of this article (doi:10.1186/s13287-017-0548-8) contains supplementary material, which is available to authorized users.

## Background

Gastric cancer (GC) is the fourth most common malignancy and the second leading cause of cancer-related death worldwide [1]. Despite breakthroughs in basic research and clinical management, the prognosis of patients with GC is still rather poor, with an average 5-year survival rate of around 30% [2]. Many GC patients die from tumor metastasis, relapse, and failure of chemotherapy.

Cancer stem cells (CSCs) are defined as a subpopulation of cells that possess self-renewal and differentiation ability [3]. Gastric CSCs have been isolated and identified from GC cell lines or tumor tissues using a serial of methods and surface markers [4–6]. Although increasing evidence has emerged to support the existence of gastric CSCs, less is known about their underlying regulatory mechanism [7]. Better understanding of the biology and mechanisms of CSCs might help improve the understanding of carcinogenesis, progression, and chemoresistance of GC.

E3 ubiquitin ligases are a large family of proteins that regulate the structure and activity of many target proteins [8]. Ring finger protein 43 (RNF43) is an E3 ubiquitin ligase originally found in stem cells [8] and is mutated in several kinds of carcinomas. Early findings described RNF43 as an oncoprotein highly expressed in colorectal tumors [9], whereas more recent research has supported its function as a tumor suppressor protein in pancreatic cancer [10], ovary cancer [11], hepatocellular cancer [12], and GC [13].

Recently, RNF43 was found to be commonly mutated in GC through whole-genome sequencing analysis [14]. RNF43 was also reported as a negative regulator of the Wnt signaling pathway, which is instrumental for development and tumor carcinogenesis. RNF43 inhibits Wnt signaling by directly ubiquitinating frizzled receptors and its downstream pathway [15]. RNF43 and its homolog, ZNRF3, are antagonized by binding of leucine-rich repeat-containing G-protein-coupled receptor 5 (Lgr5) and its ligand R-spondin. Lgr5 is an important Wnt upstream receptor [16] and considered one of the stem cell markers in the gastrointestinal tract and malignancies [17–19].

In this study, we examined the precise role of RNF43 in gastric CSCs and the underlying mechanism.

## Methods

### Clinical samples and cell lines

A total of 93 paired specimens (tumor and adjacent nontumor tissues at least 5 cm away from tumor) were obtained from GC patients who underwent surgery from 2010 to 2013 at the Department of General Surgery, Chinese PLA General Hospital (Beijing, China). This study was approved by the Institutional Review Board of the Chinese PLA General Hospital and all patients provided written informed consent. Clinicopathological information was extracted from patients’ medical records.

### Isolation of primary gastric tumor cells

Fresh human GC tissues were obtained immediately after resection from patients. All samples were transported to the laboratory on ice within 30 min and immediately disaggregated mechanically by scissors into 1-mm pieces. Tumor pieces were then digested by intermittent pipetting in phosphate buffer saline (PBS) solution with 1 mg/ml collagenase I and 1 mg/ml collagenase IV (Life Technologies, Waltham, MA, USA) at 37 °C for 1 h. Tumor digestion was terminated with DMEM/F12 medium containing 20% fetal bovine serum (FBS; Life Technologies).

### Tumorsphere culture and formation

Digested cells from primary gastric tumors or GC cell lines were cultured to form spheres in ultralow-attachment six-well and 24-well plates (Corning, NY, USA) at a density of 10,000 cells/ml in DMEM/F12 medium with 2% B27 (Invitrogen, CA, USA), EGF (20 ng/ml; Invitrogen), bFGF (10 ng/ml; Invitrogen), LIF (10 ng/ml; Peprotech, Hartford, CT, USA), and Hepes (Invitrogen). GC cells were cultured in DMEM/F12 medium with or without the addition of Wnt5a (200 ng/ml; R&D System), DKK-1 (100 ng/ml; R&D System), and R-spondin 1 (200 ng/ml; R&D System) to form spheres. Spheres with diameter > 100 μm were counted 7 days after being planted.

### RNA isolation and quantitative real-time PCR

Total RNA was extracted from tissue samples and cell lines using TRIzol reagent according to the manufacturer’s instructions (Invitrogen). First-strand cDNA was synthesized using HiScriptQ RT SuperMix for qPCR (Vazyme, Nanjing, China). Quantitative real-time PCR was performed using AceQ SYBR Master Mix (Vazyme, Nanjing, China) on a 7900HT system. The PCR primers used to amplify target genes were as follows: RNF43, forward 5′-CAAATTCACAGCCAGTGTGG-3′ and reverse 5′-GTCCTTTCCTTTCCCAGGAG-3′; β-actin, forward 5′-GCTCGTCGTCGACAACGGCTC-3′ and reverse 5′-CAAACATGATCTGGGTCATCTTCTC-3′; and Sox-2, forward 5′-GGATGGTTGTCTATTAACTT-3′ and reverse 5′-TCAAACTTCTCTCCCTTT-3′. β-actin was used as the reference gene. The Ct values of the samples were calculated, and the relative levels of mRNA were analyzed by the 2^–ΔΔCT^ method. Each sample was analyzed in triplicate to minimize the stochastic error.

### Construction of adenovirus encoding RNF43

The RNF43 cDNA and the green fluorescence protein gene (Ad-GFP) cloned by PCR were inserted into the pDC315-EGFP vector (purchased from Hanbio Co. Ltd, Shanghai, China) under the control of the mouse cytomegalovirus (CMV) promoter. The pDC315-X and pBHGlox E1,3 Cre plasmids were cotransfected into HEK293 cells to generate the recombinant adenoviruses. Ad-RNF43 and Ad-GFP were propagated in HEK293 cells. The propagated recombinant adenoviruses were purified and the titers of virus were concentrated up to 1 × 10^10^ plaque formation unit (PFU)/ml. The primers used in PCR for RNF43 cDNA generation were as follows: RNF43, forward 5′-CAACGAATTCATGAGTGGTGGCCACCAGCTGC-3′ and reverse 5′-ATTAGCGGCCGCTTACACAGCCTGTTCACACAGCTCC-3′.

### Immunohistochemistry and evaluation

Sections (5 μm thick) were cut from paraffin-fixed, paraffin-embedded tissues. The slides were dewaxed in xylene and rehydrated. The slides were heated in 0.01 mol/L citrate buffer (pH 6.0) in a microwave oven for 4 min at 100 °C for antigen retrieval. The slides were washed with PBS and blocked with 10% goat serum. Sections were treated with primary polyclonal rabbit antibody against RNF43 (ab129401, 1:200; Abcam, MA, USA), β-catenin (#8480, 1:200; Cell Signaling Technology, Danvers, MA, USA), and Sox-2 (#14962S, 1:100; Cell Signaling Technology) and incubated overnight at 4 °C. After washing with PBS, the sections were incubated for 30 min with biotinylated secondary antibody (ZSGB-BIO, Beijing, China) and peroxidase reactivity was visualized using a 3,30-diaminobenzidine (DAB) substrate kit (ZSGB-BIO). The slides were then counterstained with hematoxylin. The primary antibody was replaced with PBS as a negative control. The staining score evaluation was performed by two independent pathologists. Scores for intensity was as follows: 0, no staining; 1+, weak staining; 2+, moderate staining; and 3+, intense staining. The percentage score was as follows: 0, no staining of any cells; 1+, positive staining in up to 30% cells; 2+, positive staining in 31–60% cells; and 3+, positive staining in 61–100% cells. The final score was determined as the combination of these two scores, for which final score ≤ 2 was considered negative and score ≥ 3 was considered positive.

### Western blot analysis

Quantification of protein lysates was measured with a protein BCA assay Kit (Bio-Rad). Protein samples were separated by SDS-PAGE and transferred to a nitrocellulose membrane. The membranes were blocked with 5% nonfat milk in TBST (50 mmol/L Tris–HCl (pH 7.6), 150 mmol/L NaCl, 0.3% Tween 20) for 1 h at room temperature and then incubated in blocking buffer at 4 °C overnight with primary antibody: anti-RNF43 (SAB2102033, 1:200; Sigma-Aldrich, St. Louis, MO, USA), anti-β-catenin (#8480S, 1:1000), anti-RNF75 (#11922, 1:1000), anti-Cul4A (#2699, 1:1000), anti-C-myc (#5605, 1:2000), anti-TCF4 (#2565, 1:2000), and anti-β-actin (#3700, 1:5000; all Cell Signaling Technology). After washing with TBST, the membranes were incubated with horseradish peroxidase-coupled goat anti-rabbit/mouse secondary antibody (1:10,000; Easybio, Beijing, China) for 2 h at room temperature. Detection was performed with enhanced chemiluminescence according to the manufacturer’s instructions (ECL kit, Life Technologies).

### Flow cytometry analysis

Single cells digested by accutase (Life Technologies) from spheres in HBSS buffer (Life Technologies) were stained with stem cell markers for 20 min at 4 °C. Cells were then washed with HBSS buffer for analysis on BD Accuri C6 (BD Bioscience, MD, USA). The stem cell markers include PE-CY7 conjugated anti-human CD44 (#560533, BD Bioscience) and FITC-conjugated anti-human CD54 (#353107, Biolegend, San Diego, CA, USA). DAPI (Sigma-Aldrich) at a final concentration of 1 μg/ml was used to separate the live and dead cells. Raw data were analyzed with FlowJo software (Version 10.0.7; FlowJo, Ashland, OR, USA).

### Proliferation and chemoresistance assays

The Cell Counting Kit-8 (CCK-8; Dojindo, Kumamoto, Japan) was used to measure cell viability after RNF43 overexpression (OE). The cells were plated in DMEM (Invitrogen) at a density of 5000 cells per well in 96-well plates. CCK-8 assays were performed 0, 24, 48, and 72 h after infection. CCK-8 reagent (10 μl) was added to each well and the cells were incubated for 1 h at 37 °C. The OE and control cells were treated with chemotherapy reagent including 2.5 μg/ml 5-fluorouracil (5-Fu) (Sigma-Aldrich), 0.25 μg/ml oxaliplatin (Sigma-Aldrich), and DMSO as control. The medium for each well was replaced by normal DMEM after 72 h of treatment. Viable cell counts were estimated by measuring the optical density at 450 μm.

### Cell apoptosis assay

Cell apoptosis was measured 48 h after treating with 5-Fu (1 μg/ml) or oxaliplatin (2.5 μg/ml) using flow cytometry with the APC-Annexin V Apoptosis Detection Kit (Biolegend) according to the manufacturer’s instructions. Briefly, HGC-27 and NCI-87 cells were harvested after treatment, then washed with HBSS, resuspended in 200 μl binding buffer, and incubated with 5 μl Annexin V-APC and 10 μl PI for 20 min at room temperature. Subsequently, the number of stained cells was assessed with a flow cytometer (Accuri C6; BD Bioscience).

### In-vivo subcutaneous xenograft assay

Suspensions of RNF43-overexpressing or control cells (5 × 10^6^ cells) mixed with matrigel (Corning) at a 1:1 ratio (200 μl per mouse) were injected into the rear flank of 4-week-old male *NOD;Scid;ll2rg* ^*–/–*^ (NSG) mice to establish the xeno-transplant tumors. Tumor sizes were measured using a vernier caliper every 2 days and the tumor volume was calculated with the formula:

$$ V = 0.5 \times \mathrm{length} \times {\mathrm{width}}^2. $$

Once xenograft tumors reached about 1000 mm^3^ after 6–8 weeks, mice were sacrificed and tumors were dissected. Tumors were immediately fixed by formalin for 24 h and then embedded in paraffin. Immunohistochemical staining of RNF43 and β-catenin was performed in xenograft tumor slides according to the standard procedure already described.

### Statistical analysis

SPSS version 19.0 (SPSS Inc., Chicago, IL, USA) was applied for all statistical analysis. The results of experiments compromising two groups were analyzed using a two-tailed Student’s *t* test. Pearson’s χ^2^ test was applied to assess the various clinicopathological characteristics as a function of RNF43 expression determined by immunohistochemical analysis. Cumulative survival curves were drawn using the Kaplan–Meier method. The difference between the curves was analyzed using the log-rank test. *P* < 0.05 was considered statistically significant.

## Results

### RNF43 expression in GC

**Fig. 1:**
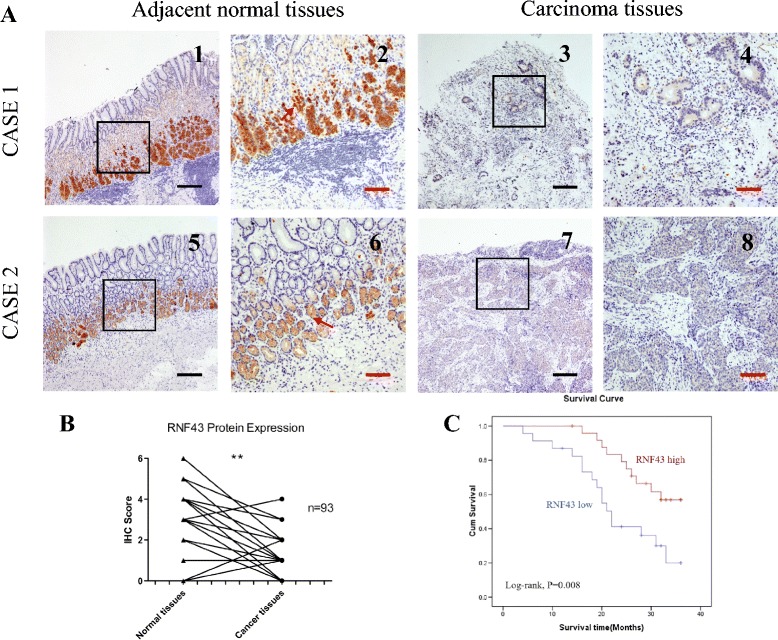
RNF43 expression is decreased in GC. **a** Representative immunohistochemical staining of RNF43 in gastric carcinoma and adjacent normal tissues. Staining of RNF43 in two pairs of matched GC tissues and their corresponding adjacent normal tissues (*1–4* for case 1; *5–8* for case 2). *Scale bar*: 10 μm (*black*) and 25 μm (*red*). **b** Immunohistochemical scores of RNF43 in tumor and adjacent tissues (*P* < 0.05). **c** Kaplan–Meier analysis indicated a correlation between RNF43 downregulation and poorer overall survival rates in GC patients (log-rank test, *P* = 0.008) (Color figure online). *IHC* immunohistochemistry

We first examined RNF43 protein expression in GC tumor tissues and normal tissues by immunohistochemistry. We detected a trend for decreased RNF43 expression in cancer tissues compared with the corresponding normal tissues (*P* < 0.01; Fig. 1a). RNF43 expression was observed in the cytoplasm and nucleus of normal epithelial cells, and the positive expression was more often detected in the lower part of the gastric epithelial layer (Fig. 1a).

Furthermore, we also detected RNF43 expression in other cancer types and their adjacent normal tissues using the same IHC staining method. The results showed that the expression of RNF43 was decreased compared with adjacent normal tissues in colon cancer, while in lung and ovarian cancer the expression in cancer tissues and normal tissues did not exhibit a significant difference (Additional file 1: Figure S1).

### Correlation of RNF43 expression with clinical variables

The association of RNF43 protein expression with the major clinicopathological features of 93 GC cases is presented in Table 1. Decreased RNF43 expression was found to be significantly associated with distant metastasis (*P* = 0.03), TNM stage (*P* = 0.033), and Lauren classification (*P* = 0.01). Moreover, lack of RNF43 expression could predict poorer overall survival of GC patients (log-rank test, *P* = 0.008; Fig. 1c). RNF43-negative patients exhibited a shorter survival time (median months, mean 21.7 ± 9.2 months) than RNF43-positive patients (mean 28.9 ± 6.8 months).

**Table 1:** Clinico-pathological variables and the expression of RNF43 in total gastric cancer patients

Characteristic	RNF43-positive	RNF43-negative	*P* value
Age (years)			
<50	14	19	0.694
>50	28	32	
Gender			
Male	20	35	0.761
Female	15	23	
Depth of invasion			
T1	1	3	0.966
T2	10	22	
T3	13	24	
T4	7	13	
Lymph node metastasis			
N0	12	8	0.075
N1	13	31	
N2	5	15	
N3	3	6	
Distant metastasis			
Negative	35	37	0.030^C^*
Positive	4	17	
pTNM stage			
I	9	10	0.033*
II	12	14	
III	4	23	
IV	5	16	
Lauren classification			
Intestinal	12	48	0.01*
Diffuse	15	18	

*C* continuity correction

*Statistically significant (*P* < 0.05)

### RNF43 expression in GC stem-like cells

**Fig. 2:**
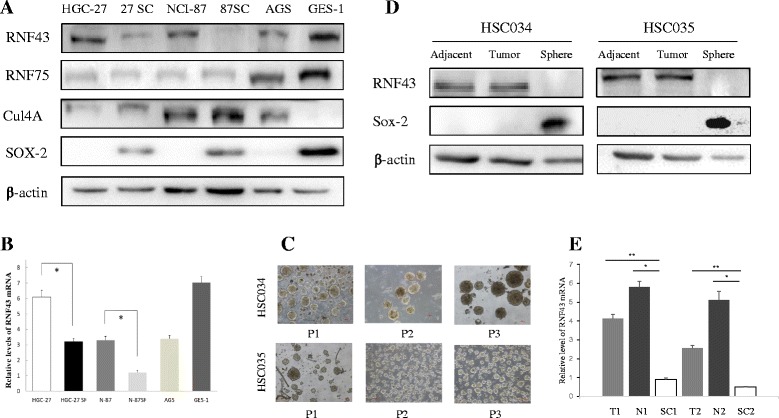
Decreased expression of RNF43 protein in GC stem-like cells. **a** Western blot assay of RNF43, RNF75, Cul4a, and Sox-2 protein in cancer cells and HGC-27 and N87 GC stem-like cells. **b** qPCR of RNF43 mRNA expression in cancer cells and CG stem-like cells. Results represent mean ± SD of three independent experiments. **c** Representative images of primary tumorsphere cells (*upper*, HSC034; *bottom*, HSC035). Most spheres were larger than 100 μm in diameter after 7 days of culture. *P* passage. **d** Western blot assay of RNF43 and Sox-2 protein in primary tumorsphere cells and corresponding cancer and adjacent normal tissues. **e** qPCR of RNF43 mRNA expression in primary tumorsphere cells and corresponding cancer and adjacent normal tissues. Results represent mean ± SD of three independent experiments. *means Statistically significant (*P* < 0.05); **means Statistically significant (*P* < 0.01)

Several different approaches have typically been adapted for the enrichment of CSCs [20, 21]. We used the ‘spheroid colony formation’ method to identify gastric cancer stem-like cells (GCSLCs) in this study, which involves culturing potential CSCs with serum-free medium containing EGF and bFGF. The growth of spherical colonies would be considered indicative of stem-like cells with self-renewal ability. Spheroid cells (SCs) were successfully obtained from HGC-27 and NCI-87 GC cell lines while AGS GC cells could not form spheres (Additional file 2: Figure S2). All tumorspheres were maintained in culture for at least 14 days and passed three times to assure self-renewal ability. Stemness associated protein Sox-2 (SRY-box 2) [22] was also measured (Fig. 2a) to confirm sphere stem-like properties. Together these results indicated that the SCs could be considered GCSLCs.

The expression of RNF43 in GC cells and GCSLCs from HGC-27 and NCI-87 cell lines was next examined by western blot assay. Compared with the adherent cells (ACs), SCs exhibited decreased expression of RNF43, and the NCI-87 SCs even showed negative expression of RNF43 (Fig. 2a). Moreover, two other members of E3 ubiquitin ligases, RNF75 and Cul4a, were measured by western blot. Although expression of  RNF75 and Cul4a in GC cells (HGC-27 and NCI-87) was different from that in GES-1 cells, the expression of these two E3 ubiquitin ligases between ACs and SCs showed no significant difference (Fig. 2a). We also performed qRT-PCR to analyze RNF43 mRNA expression in GCSLCs and ACs. The mean fold-change of RNF43 was significantly lower in GCSLCs than ACs, which was consistent with the protein levels (Fig. 2b).

To further confirm our findings, SCs were also obtained successfully from two GC patient tumor samples, HSC034 and HSC035, using the method already described. These clinical tumorspheres were maintained in culture for at least 2 months and passed three times to assure self-renewal ability (Fig. 2c). Western blot assay and qRT-PCR demonstrated that the expression of RNF43 was lost in clinical tumorspheres compared with corresponding tumor tissues and adjacent normal tissues (Fig. 2d, e).

### RNF43 OE attenuates the stem-like properties of GSCLCs

**Fig. 3:**
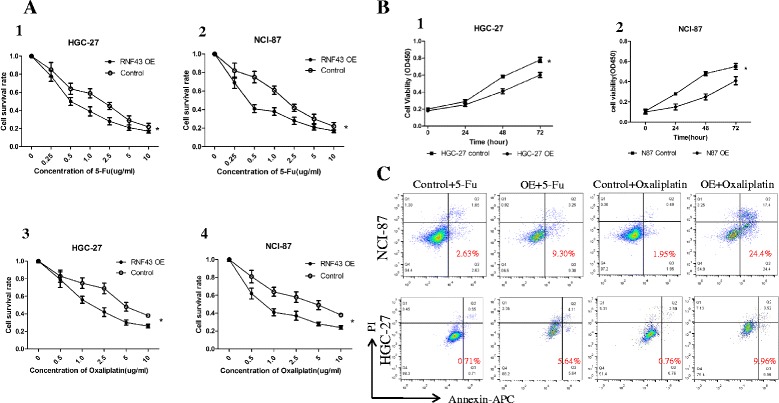
RNF43 OE affects proliferation and chemoresistance of GC cells through drug-induced apoptosis. **a** RNF43-overexpressing and control cells were treated with 5-Fu and oxaliplatin in different concentrations, and then cell viability was determined by CCK-8 assay. Results were normalized against the blank group (mean ± SD, *n* = 3). **b** CCK8 assay was performed to measure cell proliferation at the indicated times. **c** Cell apoptosis induced by 5-Fu and oxaliplatin was evaluated using Annexin V/propidium iodide-double staining. Early and late apoptotic cells were combined as Annexin V-positive cells that were considered criterion to calculate the percentage of cell apoptosis. *5-Fu* 5-fluorouracil, *OE* overexpression

Giving the finding that RNF43 expression was decreased in GC cell lines and GCSLCs, we next constructed a recombinant adenovirus carrying the RNF43 gene (Ad-RNF43). HGC-27 and NCI-87 cells were infected with Ad-RNF43 and the negative control Ad-EGFP adenovirus (Additional file 3: Figure S3) and the OE efficiency was confirmed by western blot assay. We examined the cell viability of RNF43 OE cells and control groups using CCK-8 assays and found that RNF43 OE significantly suppressed cell proliferation compared with control groups in a time-dependent manner (*P* < 0.05; Fig. 3b).

One of the important features of CSCs is resistance to chemotherapy, which could be attributed to the heterogeneity of cancer cells differentiated from CSCs. We examined the chemoresistance ability of RNF43 OE and control groups to commonly used chemotherapy drugs in GC, such as 5-Fu and oxaliplatin, with CCK-8 assays. RNF43 OE cells from HGC-27 and N87 cells showed significantly lower sensitivity with both chemotherapy drugs than control cells (*P* < 0.01) (Fig. 3a). Under certain concentrations of chemotherapy, HGC-27 OE and NCI-87 OE cell proliferation was significantly suppressed compared with control cells (Additional file 4: Figure S4). To explore the reason for RNF43 OE cells’ impaired chemotherapy resistant ability, cell apoptosis was determined by FACS. As shown in Fig. 3c, compared with the control groups, significant increases in cell apoptosis were observed in the HGC-27 OE and NCI-87 OE cells (*P* < 0.05).

**Fig. 4:**
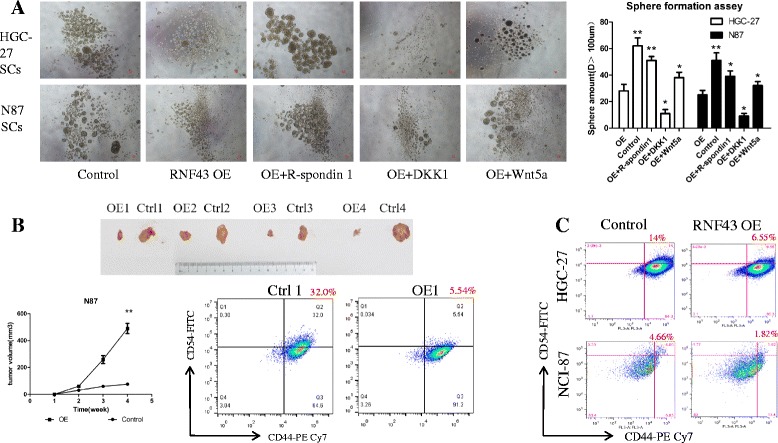
RNF43 OE attenuates stem-like properties of GCSLCs. **a** Representative images (*upper*) of RNF43 OE cells that generated smaller and fewer tumorspheres compared with control groups. Tumorsphere count (*right*) in different treatment groups, which were consistent with the trend described. Data represent mean ± SD of five independent experiments. *P < 0.05. *Scale Bar*, 50 μm. **b** In-vivo assay of the effect of RNF43 on GC stem-like cell tumorigenesis. *Upper*: xenograft tumor driven by RNF43 OE and control GC cells. *Lower left*: during 30 days after tumor cell injection, RNF43 OE cells generate smaller tumors at a lower speed compared with the control group (*P* < 0.01). *Lower right*: representative flow cytometric analysis of candidate CSC markers CD44 and CD54 for GC stem-like cells from xenograft tumor formed by RNF43 overexpressing and control cells. **c** Representative flow cytometric analysis of CD44 and CD54 for GC stem-like cells formed by control and RNF43-overexpressing cells in HGC-27 and NCI-87 cells. *OE* overexpression. **means Statistically significant (*P* < 0.01)

Sphere forming ability is also a hallmark of CSCs [20, 23]. Therefore, the sphere forming capacity of RNF43 OE cells and control cells from HGC-27 and N87 cell lines was measured by culturing cells with serum-free medium in ultralow-attachment 24-well plates. After 7 days in culture, tumorspheres with a diameter over 100 μm were counted. The RNF43 OE group formed significantly fewer spheres than in the control groups (HGC-27 control: 62 ± 5.1 spheres/well, OE: 28 ± 4.3 spheres/well, *P* < 0.01; N87 control: 51 ± 5.8 spheres/well, OE: 25 ± 3.4 spheres/well, *P* < 0.01) (Fig. 4a).

CD44 has been suggested to be the cell surface marker for gastric CSCs, but it lacks specificity due to inconsistent findings [4]. Recent studies have reported that combined CD44 and CD54 might be more accurate stem cell markers for GC [5]. To more closely determine the effect of RNF43 OE on GCSLCs, a flow cytometry assay was performed to detect CD44 and CD54 expression in RNF43 OE cells and control cells. The results showed that the percentage of CD44 and CD54 double-positive cells was lower in the RNF43 OE group compared with the control group (HGC-27, 6.3 ± 0.6% vs 14.3 ± 1.1%, respectively, *P <* 0.05; N-87, 1.8 ± 0.2% vs 4.5 ± 0.3%, respectively, *P <* 0.05%) (Fig. 4c).

CSCs are considered to exhibit high tumorigenic abilities in xenografts, which reflects their in-vivo self-renewal capability [24]. To gain insight into the role of RNF43 on tumorigenicity, equal numbers of SCs (10^4^/group) formed by RNF43 OE N-87 cells (S87 OE) and N-87 control cell lines (S87 Ctrl) were injected into two sides of rear flanks of NSG SCID mice (four mice per group) for subcutaneous xenografts. We then observed and measured the volume of subcutaneous tumors every week. The tumors derived from S87 OE mice grew much smaller and slower compared with the S87 Ctrl group (*P <* 0.01). Moreover, the proportion of CD44^+^CD54^+^ cells in tumor cells digested from xenograft was measured using cytometry assay, and showed that S87 OE xenograft tumor possessed less CD4^+^CD54^+^ cells compared with S87 Ctrl tumor, which was consistent with the findings in vitro, indicating the RNF43 OE remarkably suppresses CD44^+^CD54^+^ cancer stem-like cells (Fig. 4b).

### RNF43 negatively regulates the Wnt/β-catenin pathway

**Fig. 5:**
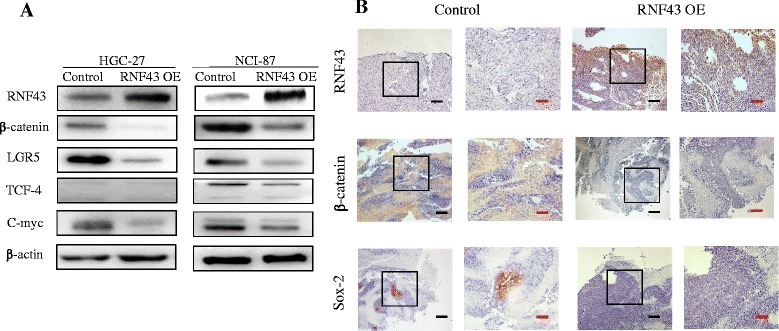
RNF43 regulates the Wnt/β-catenin pathway in GCSLCs. **a** Western blot of RNF43 expression in cancer cell lines. RNF43 OE leads to downregulation of β-catenin, a key protein in Wnt/β-catenin pathway, as well as reduction of TCF4, C-myc, and Lgr5 expression. **b** Xenograft tumor tissues were analyzed for RNF43, β-catenin, and Sox-2 expression by immunohistochemistry. *Scale bar*, 10 μm (*black*) and 25 μm (*red*) (Color figure online). OE *overexpression*

The impact of RNF43 on cancer cells varies with cancer types [25]. To determine whether RNF43 regulates the Wnt signaling pathway in GC, the key downstream proteins β-catenin, TCF-4, and C-myc were measured using western blot analysis. Decreased expression of β-catenin, TCF4, and C-myc was observed in RNF43 OE cells compared with the Ctrl group (Fig. 5a). Moreover, RNF43 markedly downregulated the expression of Lgr5 protein, which is considered an upstream activator of the Wnt signaling pathway.

Because RNF43 affected the sphere forming ability of OE cells, we next examined the effect of addition of the Wnt signaling pathway ligand R-spondin1 into sphere culture medium (Fig. 4a). After coculturing with R-spondin1, the OE cells regained their sphere forming capacity to some extent, but it was still less than in the control groups. Applying another Wnt pathway agonist (Wnt5a) also increased the expansion of tumorspheres from RNF43 OE cells. Conversely, blocking Wnt signaling by adding the Wnt antagonist DKK-1 in RNF43 OE cells yielded an opposing effect, leading to a reduced ability to from tumorspheres in vitro (Fig. 4a).

Previous in-vivo experiments revealed that RNF43 impaired tumorigenicity, and further immunohistochemical staining showed that RNF43 expression in xenograft tumor tissues had a negative correlation with β-catenin. In the RNF43 OE group, β-catenin was decreased and even lost in some regions, whereas in the control group β-catenin was relatively higher in tumor cells (Fig. 5b). In the control group, Sox-2 was expressed in part of the tissues, while in the RNF43 OE group Sox-2 was lost in the whole tissue.

## Discussion

RNF43 is located on the cell surface, and competes with Lgr5 for binding with R-spondins [8]. The presence of Lgr5 or R-spondins affects RNF43 by ubiquitinating frizzled receptors and targeting them for degradation [8, 26]. RNF43 also directly interacts with TCF4 and tethers TCF4 to the nuclear membrane, thus silencing TCF4 transcriptional activity [25].

RNF43 is mutated in several kinds of malignancies, including colorectal cancer, GC, and pancreatic cancer [25, 26]. The findings that RNF43 functions as an inhibitor against the Wnt signaling pathway are consistent with its mutation in cancers [27, 28]. However, in gastrointestinal malignancies, the results seemed to be paradoxical [9, 29]. Our study confirmed that RNF43 expression was decreased in GC tissues compared with adjacent normal tissues. Moreover, RNF43 expression was significantly lower in SCs, which are considered a form of CSCs, than in ACs. A lack of association of other E3 ubiquitin ligases and stemness indicated a unique function of RNF43 in stem-like cancer cells. Our analyses also indicated that decreased RNF43 might be associated with TNM stage, metastasis, and intestinal type cancer, and survival analysis also implied its association with poor outcome of GC patients.

Our functional studies also showed that RNF43 inhibited cell growth and chemotherapy resistance in vitro, which are also regarded as hallmarks for CSCs. The chemotherapy-induced apoptosis in RNF43 OE cells might account for the impaired proliferation and drug-resistance ability. Assays for other distinctive features for CSCs, such as tumorsphere forming ability in vitro and xenograft assays, also demonstrated that RNF43 OE would undermine self-renewal ability of GCSLCs. In sphere culture, addition of the Wnt signaling pathway activators, R-spondin1 and Wnt 5a, could reverse this trend to some extent. Stem cell surface marker analysis could reveal cells with stem cell-like properties, and our results showed that stem cell markers were significantly decreased in the RNF43 OE group both in vitro and in vivo. In xenograft tumors, RNF43 OE was correlated with decreased expression of β-catenin and Sox-2, indicating that RNF43 would alter the Wnt/β-catenin pathway and stemness in vivo*.*

Adenoviruses are small, single-strand DNA viruses that have been introduced in increasing clinical applications as a result of their stable gene delivery efficacy, high safety profile, and low pathogenicity [30]. Despite the growing number of studies on CSCs, there are still few therapies directly targeting CSCs [31]. Because the Ad-RNF43 we used in this study significantly inhibited viability of cells with low RNF43 expression compared with high RNF43 expression, our results might indicate a potential therapeutic application for Ad-RNF43 in GC CSC treatment.

The limitations of our study still need to be addressed. First, the SCs derived from primary GC tumors are too fragile to undergo virus infection, and thus the OE study was performed in established GC cell lines, which restrains the reliability of the conclusions. Second, surface marker analysis demonstrated that the stem-like cancer cells only account for a small population of the total cells, which might result in an inaccuracy of the effect of RNF43 on CSCs. This phenomenon might arise from the fact that CSCs could generate differentiated daughter cells [32]. Takaishi et al. [4] also found that CD44-positive GC cells were able to generate CD44-negative cells during culture, which retain self-renewal capacity in just a small portion of cells. Third, due to the varied expression of RNF43 in different cancer cell lines, whether downregulation of RNF43 in highly expressed GC cell lines would render stemness to these cancer cells remains unclear, and needs more study to elucidate.

## Conclusion

Our results showed that RNF43 is negatively correlated with GC patient clinical outcome and that the expression of RNF43 was significantly lower in GCSLCs than in ACs and normal mucosal cells. Furthermore, adenovirus-induced RNF43 OE inhibited tumor growth and stem cell-like phenotype through the canonical Wnt/β-catenin pathway both in vitro and in vivo. These findings suggest that the clinical value of adenovirus-mediated RNF43 targeting CSCs in GC is worth further exploration.

## Additional files

Additional file 1: Figure S1. IHC images of RNF43 in colon cancer, ovarian cancer, lung cancer, and their corresponding normal tissues (*Scale bar*, 10 μm (*black*)). (PDF 187 kb)

Additional file 2: Figure S1. Gastric cancer cell lines in tumorsphere forming medium after 7 days (*Scale bar*, 50 μm). (PDF 186 kb)

Additional file 3: Figure S3. Infection efficiency of Ad-RNF43 in gastric cancer cells: *left*, observation under a regular microscope; *right*, observation of the same field under a fluorescence microscope (*Scale Bar*, 50 μm). (PDF 72 kb)

Additional file 4: Figure S4. CCK-8 assay of HGC-27 and NCI-87 treating with 5-Fu (1 μg/ml) and Oxaliplatin (2.5 μg/ml). (PDF 411 kb)

## Abbreviations

AC: Adherent cell
bFGF: Basic fibroblast growth factor
CSC: Cancer stem cell
EGF: Epidermal growth factor
GC: Gastric cancer
GCSLC: Gastric cancer stem-like cell
IHC: Immunohistochemistry
Lgr5: Leucine-rich repeat-containing G-protein-coupled receptor 5
OE: Overexpression
RNF43: Ring finger protein 43
SC: Sphere cell
