# Supplementary material for: Ring finger protein 43 associates with gastric cancer progression and attenuates the stemness of gastric cancer stem-like cells via the Wnt-β/catenin signaling pathway
Source: Stem Cell Res Ther. 2017 Apr 26;8:98. doi: 10.1186/s13287-017-0548-8 (PMC5406878; doi:10.1186/s13287-017-0548-8)
Supplement: Supplementary file 1 — IHC images of RNF43 in colon cancer, ovarian cancer, lung cancer, and their corresponding normal tissues (Scale bar, 10 μm (black)). (PDF 187 kb) [file 13287_2017_548_MOESM1_ESM.pdf]

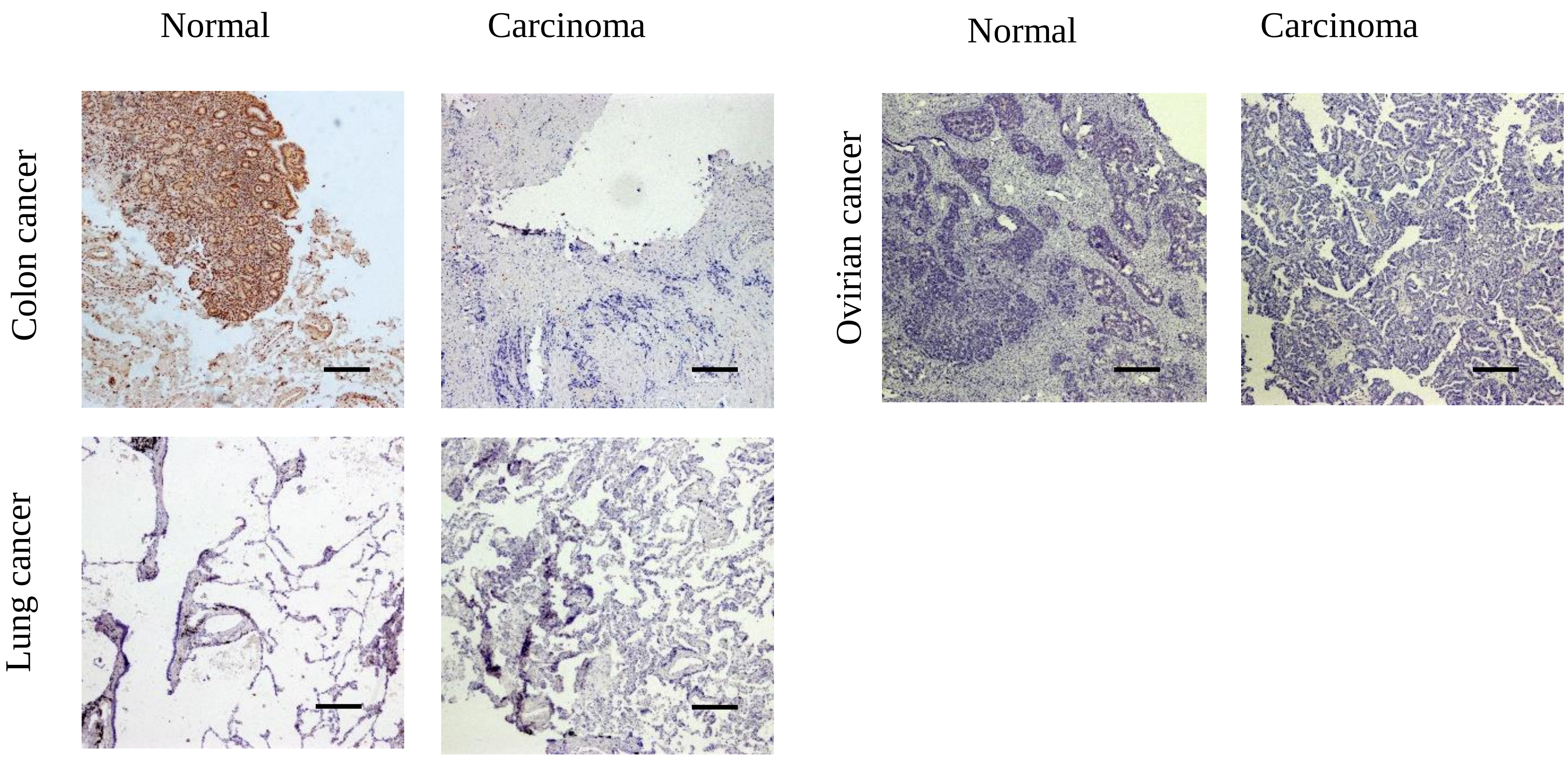

Fig. S1 IHC images of RNF43 in colon cacner, ovrian cancer, lung cancer and their corresponding normal tissues. (Scale bar, 10μm in black)
