# Supplementary material for: Ring finger protein 43 associates with gastric cancer progression and attenuates the stemness of gastric cancer stem-like cells via the Wnt-β/catenin signaling pathway
Source: Stem Cell Res Ther. 2017 Apr 26;8:98. doi: 10.1186/s13287-017-0548-8 (PMC5406878; doi:10.1186/s13287-017-0548-8)
Supplement: Supplementary file 2 — Gastric cancer cell lines in tumorsphere forming medium after 7 days (Scale bar, 50 μm). (PDF 186 kb) [file 13287_2017_548_MOESM2_ESM.pdf]

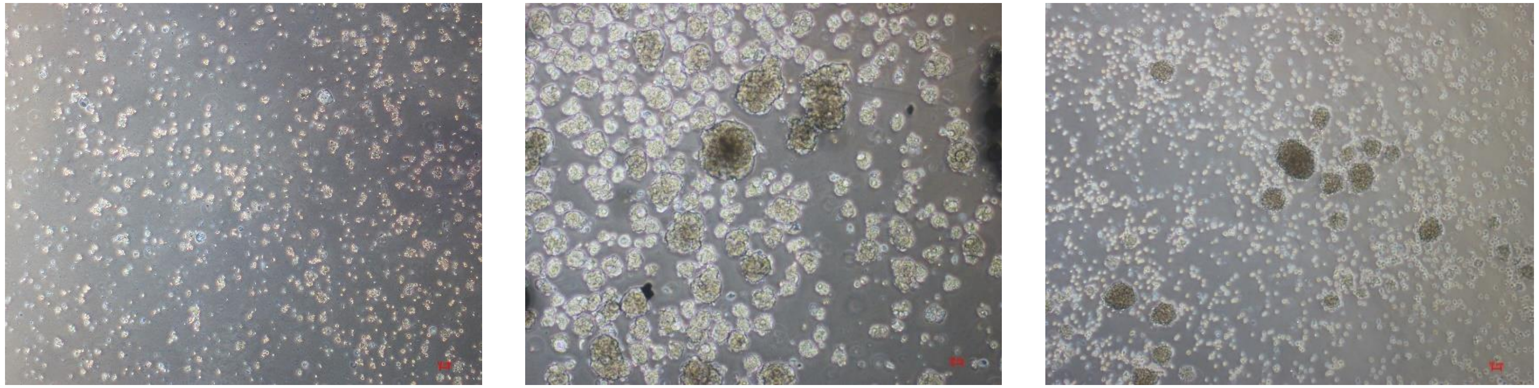

AGS(7th Day) N87（7th Day） HGC-27 （7th Day）

Fig. S2 Gastric cancer cell lines in tumor sphere forming medium after 7 days (Scal bar, 50µm)
