# Supplementary material for: Ring finger protein 43 associates with gastric cancer progression and attenuates the stemness of gastric cancer stem-like cells via the Wnt-β/catenin signaling pathway
Source: Stem Cell Res Ther. 2017 Apr 26;8:98. doi: 10.1186/s13287-017-0548-8 (PMC5406878; doi:10.1186/s13287-017-0548-8)
Supplement: Supplementary file 3 — Infection efficiency of Ad-RNF43 in gastric cancer cells: left, observation under a regular microscope; right, observation of the same field under a fluorescence microscope (Scale Bar, 50 μm). (PDF 72 kb) [file 13287_2017_548_MOESM3_ESM.pdf]

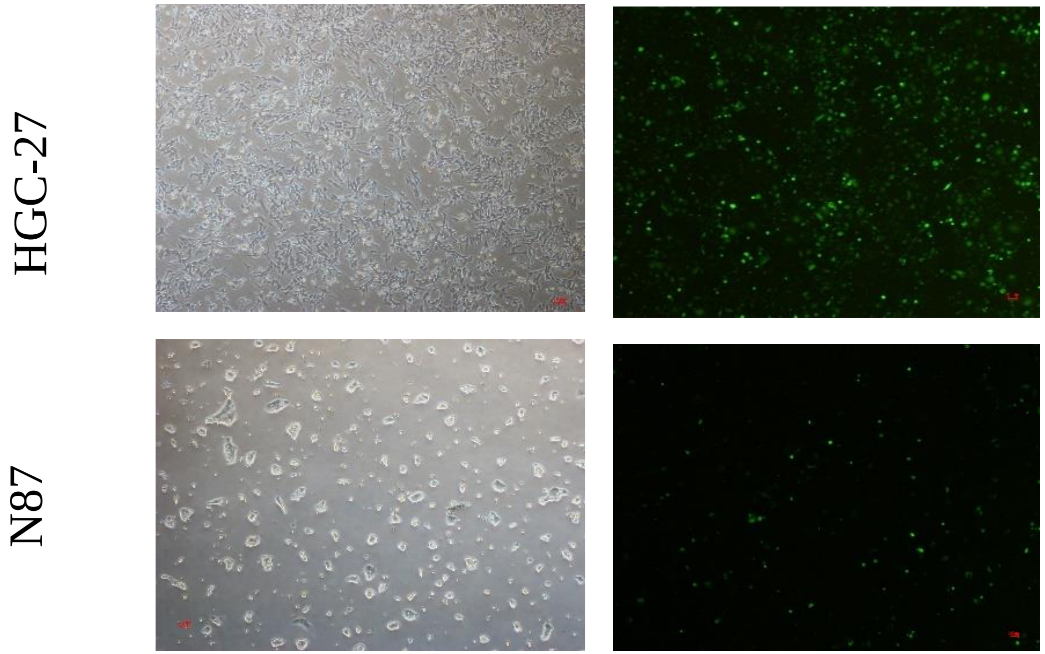

Fig. S3 Infection efficiency of Ad-RNF43 in gastric cancer cells. Left, observation under a regular microscope; right, observation of the same field under a fluorescence microscope. (Scale Bar, 50μm)
