# Supplementary figures and images for: Ring finger protein 43 associates with gastric cancer progression and attenuates the stemness of gastric cancer stem-like cells via the Wnt-β/catenin signaling pathway
Source: Stem Cell Res Ther. 2017 Apr 26;8:98. doi: 10.1186/s13287-017-0548-8 (PMC5406878; doi:10.1186/s13287-017-0548-8)

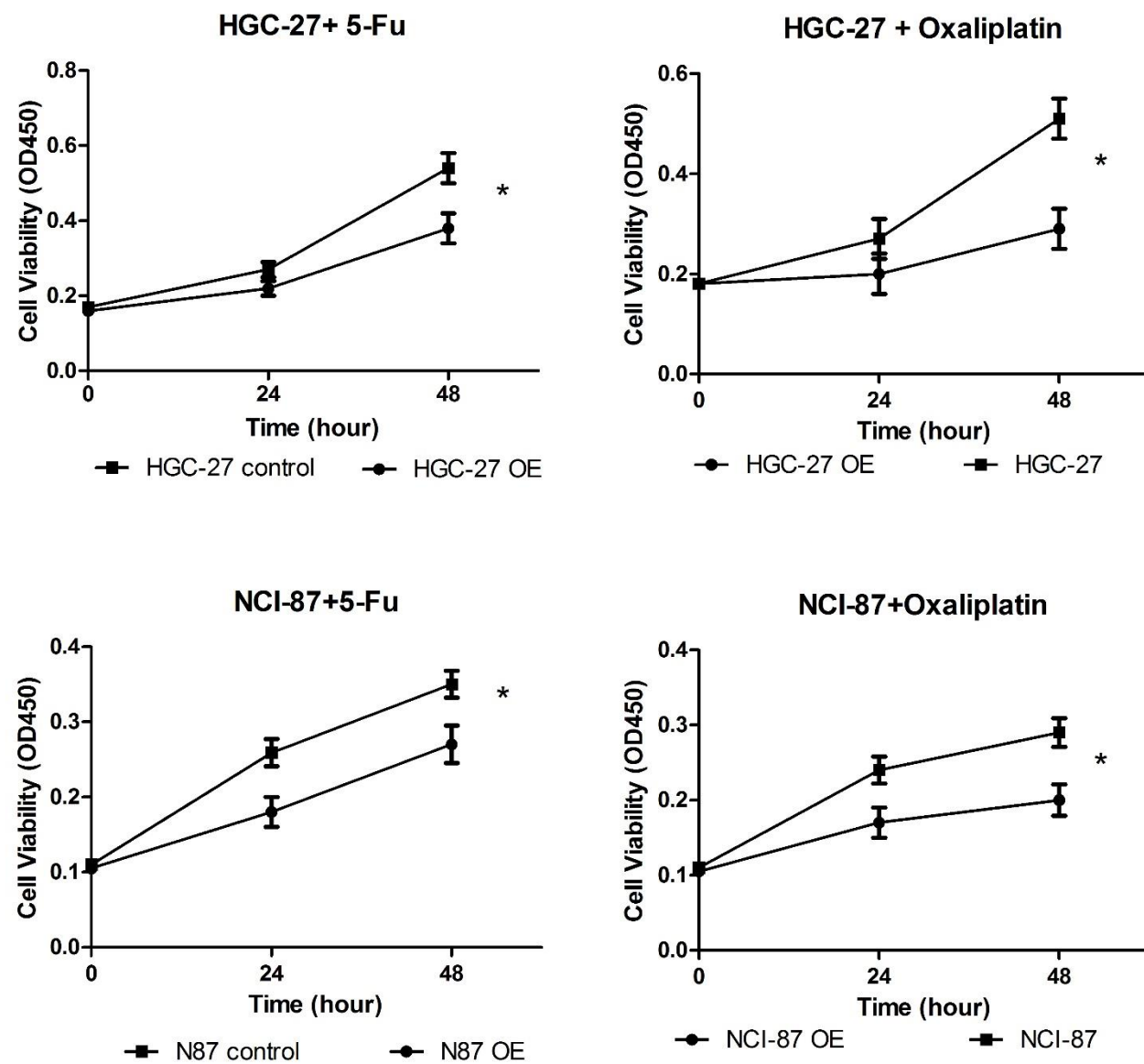

Fig. S4 CCK-8 assay of HGC-27 and NCI-87 treating with 5-Fu(1 $\mu$ g/ml) and Oxaliplatin (2.5 $\mu$ g/ml).

Supplement: Supplementary file 4 — CCK-8 assay of HGC-27 and NCI-87 treating with 5-Fu (1 μg/ml) and Oxaliplatin (2.5 μg/ml). (PDF 411 kb) [file 13287_2017_548_MOESM4_ESM.pdf]
